# T Cells of Different Developmental Stages Differ in Sensitivity to Apoptosis Induced by Extracellular NAD

**DOI:** 10.1080/10446670310001593514

**Published:** 2002-12

**Authors:** Friedrich Haag, Dunja Freese, Felix Scheublein, Wiebke Ohlrogge, Sahil Adriouch, Michel Seman, Friedrich Koch-Nolte

**Affiliations:** 1 Institute of Immunology University Hospital Martinistr. 52 Hamburg D-20246 Germany; 2 Laboratoire d'Immunodifferenciation, EA 1556 Université Denis Diderot Paris 7 CP Tour 54 2 place Jussieu Paris Cedex 05 75251 France

## Abstract

Extracellular nucleotides such as ATP and NAD can profoundly affect the functions of lymphocytes, macrophages, and other cells. We have recently shown that extracellular NAD induces rapid apoptosis in naive T cells by a mechanism involving the ADP-ribosylation of cell surface molecules. In the present paper, we describe that T cells of different developmental stages differ in their sensitivity to NAD-induced apoptosis. Thymocytes were less susceptible than peripheral lymph node T cells, and freshly activated cells were more resistant than resting cells. Sensitivity to NAD-induced apoptosis generally correlated with expression of the ADP-ribosyltransferase ART2.2, which is not expressed on thymocytes and shed from peripheral T cells upon activation. Our findings suggest that NAD-induced apoptosis does not play a role during thymic selection of T cells, but rather may play a role by preventing the activation of unwanted bystander T cells during an immune response, and thus may participate in the control of autoimmunity.


*Developmental Immunology*, December 2002 Vol. 9 (4), pp. 197–202

Taylor & Francis healthsciences

### T Cells of Different Developmental Stages Differ in Sensitivity to Apoptosis Induced by Extracellular NAD*

FRIEDRICH HAAG[a,†], DUNJA FREESE[a], FELIX SCHEUBLEIN[a], WIEBKE OHLROGGE[a], SAHIL ADRIOUCH[b], MICHEL SEMAN[b] and FRIEDRICH KOCH-NOLTE[a]

[a]*Institute of Immunology, University Hospital, Martinistr. 52, D-20246 Hamburg, Germany;* [b]*Laboratoire d'Immunodifferenciation, EA 1556, Université Denis Diderot, Paris 7, CP7124, Tour 54, 2 place Jussieu, 75251 Paris Cedex 05, France*

Extracellular nucleotides such as ATP and NAD can profoundly affect the functions of lymphocytes, macrophages, and other cells. We have recently shown that extracellular NAD induces rapid apoptosis in naive T cells by a mechanism involving the ADP-ribosylation of cell surface molecules. In the present paper, we describe that T cells of different developmental stages differ in their sensitivity to NAD-induced apoptosis. Thymocytes were less susceptible than peripheral lymph node T cells, and freshly activated cells were more resistant than resting cells. Sensitivity to NAD-induced apoptosis generally correlated with expression of the ADP-ribosyltransferase ART2.2, which is not expressed on thymocytes and shed from peripheral T cells upon activation. Our findings suggest that NAD-induced apoptosis does not play a role during thymic selection of T cells, but rather may play a role by preventing the activation of unwanted bystander T cells during an immune response, and thus may participate in the control of autoimmunity.

*Keywords*: T cells; Apoptosis; Extracellular nucleotides; ADP-ribosylation; Mono(ADP-ribosyl)-transferases

### INTRODUCTION

In order to survive, organisms must maintain a tight control over the numbers and receptor specificities of immune cells. The regulation of cell death and survival is central to this task. Throughout their lifespan, T lymphocytes are continually subject to processes that may result in their death (van Parijs and Abbas, 1998). Regulated cell death may result either from the withdrawal of survival signals (death by neglect) or from the receipt of death signals (actively induced cell death). A prototype of this latter mechanism is the induction of apoptosis via signals transmitted through the TNFR family of death receptors. We have recently reported that micromolar concentrations of extracellular NAD induce apoptosis in T cells via a mechanism involving the ADP-ribosylation of cell surface molecules (Adriouch *et al.*, 2001). Besides the availability of ecto-NAD, cell death in this model required the presence of a cell surface mono(ADP-ribosyl)transferase (ART), as well as one or more unknown downstream effector molecules, presumably targets of ADP-ribosylation and/or components of the signal transduction machinery.

ARTs post-translationally modify proteins by transferring an ADP-ribose moiety from NAD to specific amino acids, e.g. arginine residues, of target proteins. ARTs have well-characterized regulatory functions in the prokaryotic world (Ludden, 1994). Several prokaryotic ARTs are secreted and function as toxins that exert potent effects on mammalian cells by inactivating key proteins in their target cells (Koch-Nolte and Haag, 1997). In mammals, a family of toxin-related extracellular ARTs has been identified that are expressed either as GPI-anchored or secreted molecules by different cell types (Koch-Nolte and Haag, 1997; 1998; Glowacki *et al.*, 2002).

ARTs have been implicated in T cell differentiation and the regulation of immune function. Rat ART2, formerly known as RT6, is a marker for mature T cells (Thiele *et al.*, 1997). A subset of ART2+ T cells exerts a regulatory function in the BB rat model for autoimmune diabetes mellitus (Greiner *et al.*, 1986; 1987). T cells from diabetes-prone BB rats show reduced expression of ART2, and transfer of ART2+ T cells from diabetes-resistant rats prevents the disease. The mouse carries two *Art2* genes, and, as in the rat, expression of the corresponding gene products, ART2.1 and ART2.2, is restricted to mature T cells (Prochazka *et al.*, 1991; Koch-Nolte *et al.*, 1999). NAD-dependent ADP-ribosylation of cell surface proteins has been shown to inhibit the proliferation

*Presented at the Proceedings of the 4th Germinal Center Conference, June 2002, Groningen, The Netherlands.

†Corresponding author. Tel.: +49-40-428034595. Fax: +49-40-428034243. E-mail: haag@uke.uni-hamburg.de

ISSN 1044-6672 print © 2002 Taylor & Francis Ltd
DOI: 10.1080/10446670310001593514

and cytotoxic effector functions of CTL lines *in vitro* (Wang *et al.*, 1994; 1996), and to inhibit proliferation (Okamoto *et al.*, 1998) and induce apoptosis (Adriouch *et al.*, 2001; Liu *et al.*, 2001) in primary T cells.

In this report, we examine the susceptibility of T cells of different developmental stages to NAD-induced apoptosis. Based on the finding that mature resting T cells represent the cell population most sensitive to NAD-induced cell death, the hypothesis is presented that ART-mediated cell death could play a role in the control of autoimmunity by preventing the activation of bystander cells during an immune reaction.

### MATERIALS AND METHODS

### Materials

Chemicals were from Sigma (Deisenhofen, Germany). AnnexinV-FITC, anti-CD3-PE, anti-rat-Ig-PE, and anti-CD3-, -CD28-, and -CD95-antibodies were from Pharmingen (Heidelberg, Germany). ART2.2-specific antibody Nika102 has been described (Koch-Nolte *et al.*, 1999).

### Animals and Preparation of Cells

Six to eight week-old BALB/cByJ mice were obtained from Charles River (Sulzfeld, Germany). Thymi and lymph nodes were isolated from sacrificed animals, and single cell suspensions were prepared and processed for flow cytometry on a FACScan (Becton Dickinson) as described previously (Adriouch *et al.*, 2001). Where indicated, T cells were enriched by depletion of B cells using magnetic cell separation with Dynabead-immobilized goat anti-mouse IgG (Dynal, Hamburg, Germany, 4–6 beads/cell).

### Assay for Phosphatidylserine (PS) Exposure

Apoptotic and necrotic cells were stained with AnnexinV-FITC and propidium iodide essentially as described previously (Adriouch *et al.*, 2001). In brief, following treatment with the indicated concentrations of extracellular ATP or bzATP at 37°C, cells were washed in RPMI medium supplemented with 2 mM $CaCl_2$, and were then stained in this medium for 20 min on ice with FITC-conjugated AnnexinV (1 μg/ml) and propidium iodide (10 μg/ml) or PE-conjugated anti-mouse IgG.

### RESULTS

### Ecto-NAD Induces Rapid Apoptosis of T Cells by a Mechanism Involving ADP-ribosylation

In a previous report, we showed that treatment with extracellular NAD induces rapid T cell death by apoptosis (Adriouch *et al.*, 2001). This is evidenced first by exposure of PS on the outer leaflet of the plasma membrane, by failure to exclude propidium iodide (Fig. 1a), and ultimately by fragmentation of DNA (Adriouch *et al.*, 2001). Induction of apoptosis by NAD is rapid, as PS-exposure evidenced by AnnexinV staining was observed as early as 0.5 min after exposure to NAD (Fig. 1b).

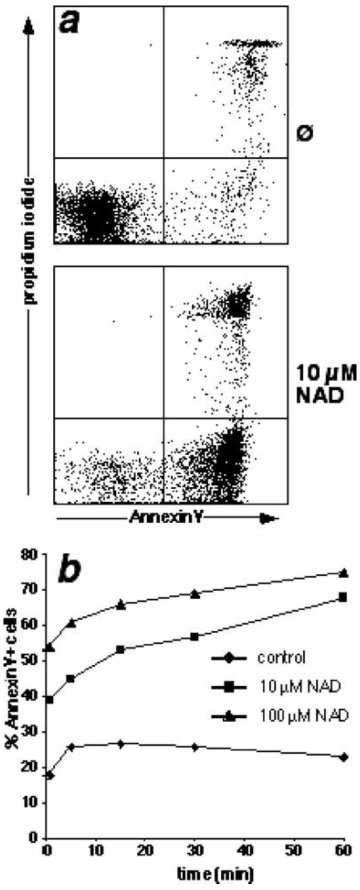

FIGURE 1 Extracellular NAD induces rapid phosphatidylserine exposure and cell death in T lymphocytes. Purified lymph node T cells were incubated with the indicated concentrations of NAD at 37°C for 2 h (a) or the indicated times (b) before staining with annexinV-FITC and propidium iodide.

### Thymocytes and Peripheral T Cells Differ in Sensitivity to NAD-induced and CD95-mediated Apoptosis

To examine the sensitivity of T cells at different stages of development to ecto-NAD-induced apoptosis, thymocytes and peripheral lymph node cells were incubated for 2 h with different concentrations of NAD or with 2 μg of anti-CD95 antibody, and assayed for PS exposure by AnnexinV staining (Fig. 2). Lymph node cells responded to ecto-NAD in a dose-dependent manner, but were resistant to treatment with anti-CD95 antibody. Thymocytes, by contrast, became AnnexinV-positive in response to anti-CD95, but remained resistant to the effects of even 100 μM ecto-NAD. Concomitant staining for AnnexinV-binding and expression of CD3 showed that among

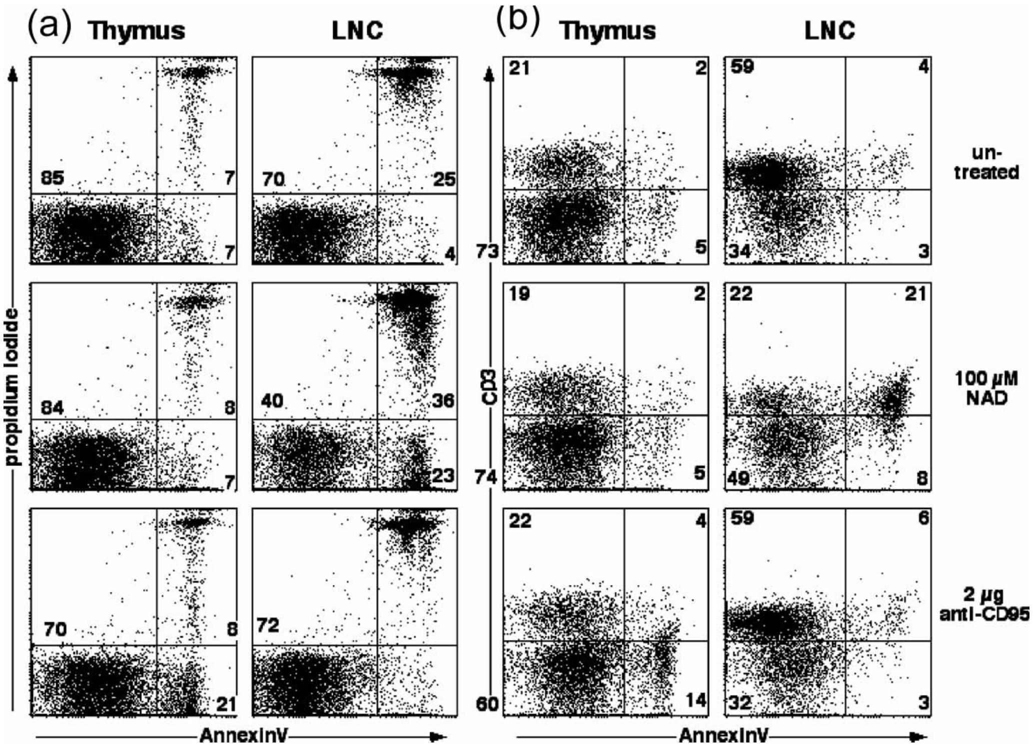

FIGURE 2 Differential sensitivity of thymocytes and lymph node cells to NAD and anti-CD95 antibodies. Thymocytes and total lymph node cells were incubated for two hours at 37°C with NAD or anti-CD95 antibodies before staining with annexinV-FITC and propidium iodide (a) or anti-CD3 antibodies (b).

peripheral lymph node cells only T cells are sensitive to ecto-NAD-induced apoptosis. In the thymus, neither immature CD3lo nor the more mature CD3hi cells responded to ecto-NAD, while CD95-mediated apoptosis was observed within the CD3lo population (Fig. 2b).

### Activated T Cells Shed ART2 and Show Decreased Sensitivity to Ecto-NAD-induced Apoptosis

We have previously reported that T cells release cell surface ART2 upon stimulation with PMA by a metalloproteinase-mediated mechanism (Kahl *et al.*, 2000). To examine whether T cells also become resistant to NAD-induced apoptosis upon activation, purified lymph node T cells were incubated for 2 h with polyclonal T-cell stimulators before treatment with NAD. Treatment with either phorbol myristate acetate (PMA) or a combination of anti-CD3 and anti-CD28 antibodies caused a marked reduction in the level of cell surface ART2.2 compared to untreated cells (Fig. 3a). In addition, both treatments also led to increased resistance to NAD-induced apoptosis, as evidenced by reduced staining with AnnexinV and propidium iodide compared to control cells (Fig. 3b).

### DISCUSSION

The maintenance of homeostasis in the immune system and the focusing of immune reactions on appropriate targets require the coordinated interplay of regulatory mechanisms, many of which involve programmed cell death (van Parijs and Abbas, 1998). We have recently identified a new death signal for lymphocytes: the exposure to extracellular NAD (Adriouch *et al.*, 2001). Aim of the present study was to examine the sensitivity of T cells to NAD-induced apoptosis during different stages of development.

In naive peripheral T cells, exposure to ecto-NAD causes exposure of PS and permeabilization to propidium iodide. PS-exposure, as evidenced by AnnexinV-binding, is an early indicator of apoptosis in many cell types (Bossy-Wetzel and Green, 2000) although it has been reported to be reversible in some cases (MacKenzie *et al.*, 2001). In naive peripheral T cells, PS exposure is followed by failure to exclude PI and ultimately by fragmentation of DNA (Adriouch *et al.*, 2001), indicating that these cells indeed have progressed to cell death. NAD-induced apoptosis is rapid, since PS exposure was detected as early as 0.5 min following treatment with NAD. Several lines of evidence suggest that the pro-apoptotic effects of NAD are mediated via the ADP-ribosylation of cell surface molecules. First, among peripheral lymphocytes those cells expressing the T cell-specific ADP-ribosyl-transferase ART2 are most susceptible to NAD-induced apoptosis (Adriouch *et al.*, 2001). Further, treatment of cells with phosphatidylinositol-specific phospholipase C (PI-PLC), which removes glycosyl-phosphatidyl-inositol- (GPI-) anchored ART2 from the cell surface, renders cells resistant to NAD-induced apoptosis

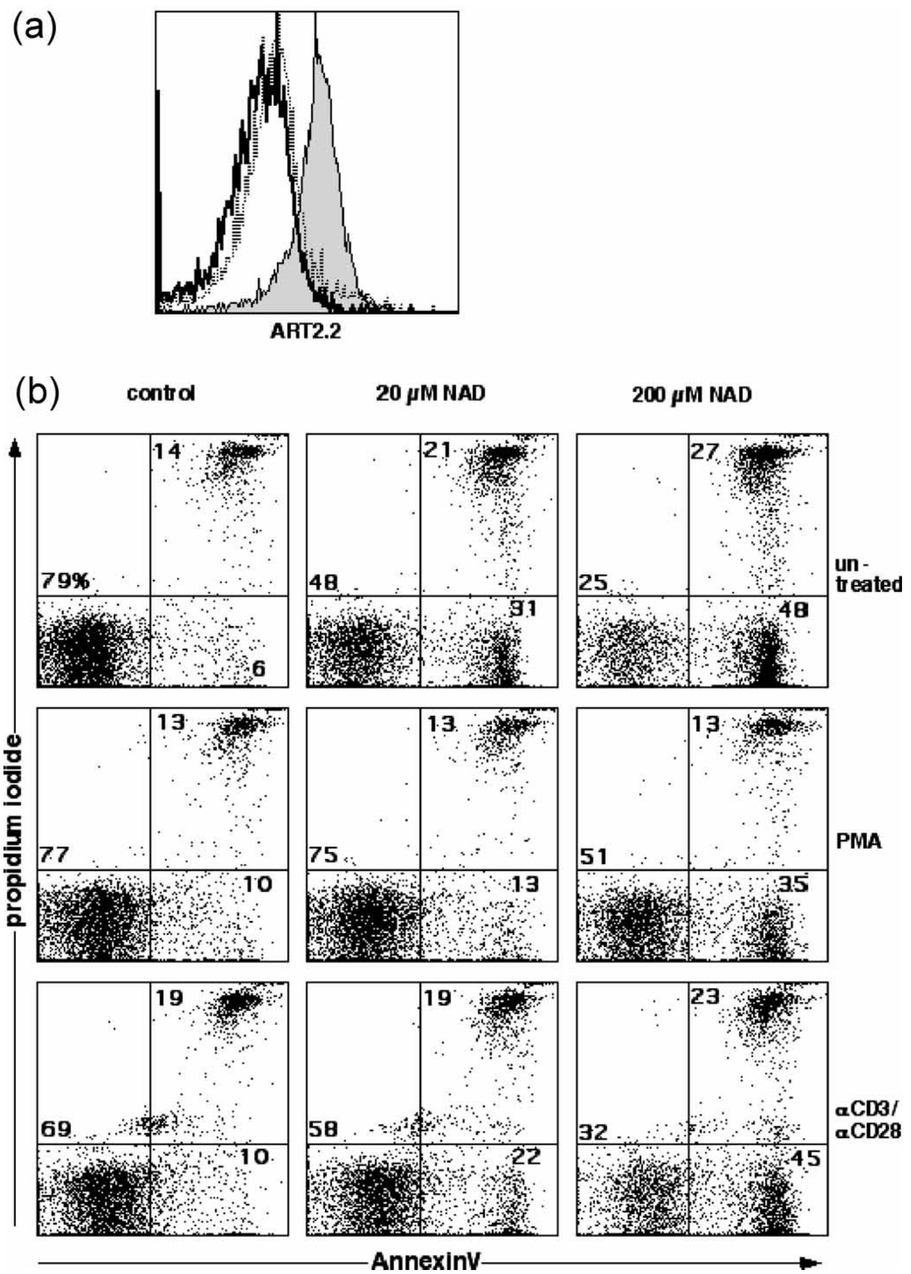

FIGURE 3 Activated T cells shed ART2.2 and become resistant to NAD-induced apoptosis. Purified lymph node T cells were incubated for 2 h at 37°C with 100ng/ml PMA or 1 μg/ml plate-bound anti-CD3/CD28 antibodies. (a) Expression of ART2.2 was analyzed by staining with Nika102 followed by anti-rat-Ig-PE. Untreated cells are represented by the shaded histogram, PMA- and anti-CD3-treated cells by bold and dotted lines, respectively. (b) Cells as in (a) were treated for 30 min at 37°C with the indicated concentrations of NAD before staining with annexinV-FITC and propidium iodide.

(Adriouch *et al.*, 2001). Moreover, NAD-induced apoptosis can be blocked by antibodies against ART2 (Adriouch *et al.*, 2001). Finally, activation of T cells, which induces shedding of ART2 (Kahl *et al.*, 2000), correlates with increased resistance to NAD-induced PS-exposure.

T cells are susceptible to programmed cell death at all stages of their development. In the thymus, regulation of apoptosis in the context of positive and negative selection of thymocytes is important for the shaping of the T cell repertoire. In the periphery, resting T cells depend on survival signals for their maintenance. After activation, withdrawal of survival signals and activation-induced cell death (AICD) serve to reduce lymphocyte numbers during the waning of an immune response and to eliminate auto-reactive T cells. Here, we examined the differential sensitivity of thymocytes as well as resting and activated T cells to NAD-induced apoptosis and compared the effects of NAD to a classical "active" death signal, triggering of the CD95 receptor by anti-CD95 antibodies. The results show that mature resting T cells represent the population most susceptible to NAD-induced apoptosis, while thymocytes are completely, and activated lymph node T cells partially resistant. Thus, susceptibility to

NAD-induced apoptosis mirrors expression of ART2 (Koch-Nolte *et al.*, 1999). This enzyme is expressed on the surface of mature T cells and shed from the surface of activated T cells by the action of a metalloproteinase (Kahl *et al.*, 2000). Interestingly, NAD-induced apoptosis does not occur within the population of mature CD3hi thymocytes, a portion of which express ART2 (Koch-Nolte *et al.*, 1999). This may be due to the absence in these cells of a downstream effector, which previous studies have shown to be necessary for ART-mediated apoptosis (Adriouch *et al.*, 2001). Resting peripheral T cells were completely resistant to CD95-mediated apoptosis under the conditions employed in our experiments. This presumably reflects a requirement for pre-activation in these cells for the assembly of the death-inducing signaling complex (DISC), necessary for the transduction of death signals via CD95. By contrast, apoptosis was readily visible within the immature CD3lo population of thymocytes, reflecting the importance of CD95 for thymocyte selection (Debatin *et al.*, 1994; Anderson *et al.*, 1996). What may be the biological role of NAD-induced cell death? Our results indicate that ART-mediated apoptosis does not contribute to thymic selection, but rather suggest that the importance of ecto-NAD as a death signal lies within the peripheral T cell compartment. Extracellular NAD differs from other "active" death signals in several respects. First of all, it induces the exposure of PS more rapidly than signaling via death receptors. Secondly, ecto-NAD as a death signal within the immune system is unique in that it selectively affects mature resting T cells. As discussed above, expression of ART2 is a pre-requisite for NAD-induced apoptosis. Naturally occurring deficiencies of ART2 (formerly designated Rt6) have been observed in several mouse and rat models for autoimmune diseases. It is thus, an attractive hypothesis that ecto-NAD-induced cell death contributes to the prevention of autoimmunity. We propose that this occurs by eliminating unwanted bystander cells that may become activated in the course of an immune reaction (Fig. 4). Under physiological conditions the concentration of extracellular NAD is low, i.e. in the nanomolar range. Micromolar concentrations of extracellular NAD, which are sufficient to trigger NAD-induced cell death, may be expected to occur either as a result of local tissue injury, as happens during inflammation, or as the result of regulated secretion mechanisms (Bruzzone *et al.*, 2001). In the scenario of an immune reaction against a pathogen, antigen-specific activated T effector cells, which themselves have shed cell surface ARTs and thus are resistant to NAD-induced cell death (Fig. 4a), cause the lysis of target cells, thereby releasing NAD into the extracellular compartment (Fig. 4b). Resting bystander cells, which are not specific for the pathogen, but which might become activated due to the multitude of stimulatory signals generated during the inflammatory response, would be eliminated following exposure to NAD and ART2-catalyzed ADP-ribosylation of cell surface target proteins (Fig. 4c).

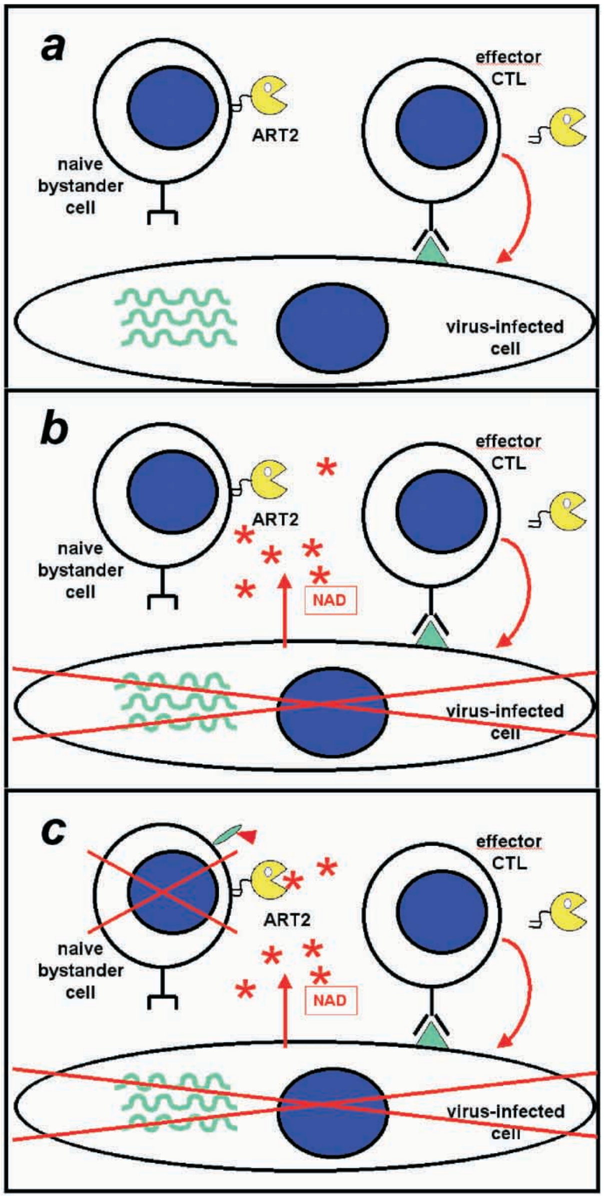

FIGURE 4 NAD-induced apoptosis may control autoimmunity by preventing the activation of bystander cells. See text for details. Extracellular NAD is denoted by red stars, ADP-ribosylation of an unknown target protein by a red triangle.

In conclusion, ecto-NAD-induced cell death provides a novel mechanism of focusing an apoptotic signal to a selected population of cells which express appropriate "receptor" molecules that are able to process the signal. These molecules include the ARTs, but also one or more as yet unknown effector molecules that are targets for ADP-ribosylation. It will be a challenge to identify these downstream effectors, and to determine whether NAD-induced cell death also operates outside of the immune system.

### *Acknowledgements*

We thank Gudrun Dubberke and Vivienne Welge for expert technical assistance. This work was supported by grant SFB 545/B9 of the Deutsche Forschungsgemeinschaft to FKN and FH, and by a grant from the Ministère de la Recherche et de la Technologie. SA is recipient of a fellowship from the Association pour la Recherche sur le Cancer.

### References

Adriouch, S., Ohlrogge, W., Haag, F., Koch-Nolte, F. and Seman, M. (2001) "Rapid induction of naive T cell apoptosis by ecto-nicotinamide adenine dinucleotide: requirement for mono(ADP-ribosyl)transferase 2 and a downstream effector", *J. Immunol.* **167**, 196–203.

Anderson, K.L., Anderson, G., Michell, R.H., Jenkinson, E.J. and Owen, J.J. (1996) "Intracellular signaling pathways involved in the induction of apoptosis in immature thymic T lymphocytes", *J. Immunol.* **156**, 4083–4091.

Bossy-Wetzel, E. and Green, D.R. (2000) "Detection of apoptosis by annexin V labeling", *Methods Enzymol.* **322**, 15–18.

Bruzzone, S., Guida, L., Zocchi, E., Franco, L. and de Flora, A. (2001) "Connexin 43 hemi channels mediate Ca2+-regulated transmembrane NAD + fluxes in intact cells", *FASEB J.* **15**, 10–12.

Debatin, K.M., Suss, D. and Krammer, P.H. (1994) "Differential expression of APO-1 on human thymocytes: implications for negative selection?", *Eur. J. Immunol.* **24**, 753–758.

Glowacki, G., Braren, R., Firner, K., Nissen, M., Kuhl, M., Reche, P., Bazan, F., Cetkovic-Cvrlje, M., Leiter, E., Haag, F. and Koch-Nolte, F. (2002) "The family of toxin-related ecto-ADP-ribosyltransferases in humans and the mouse", *Protein Sci.* **11**, 1657–1670.

Greiner, D.L., Handler, E.S., Nakano, K., Mordes, J.P. and Rossini, A.A. (1986) "Absence of the RT-6 T cell subset in diabetes-prone BB/W rats", *J. Immunol.* **136**, 148–151.

Greiner, D.L., Mordes, J.P., Handler, E.S., Angelillo, M., Nakamura, N. and Rossini, A.A. (1987) "Depletion of RT6.1+ T lymphocytes induces diabetes in resistant biobreeding/Worcester (BB/W) rats", *J. Exp. Med.* **166**, 461–475.

Haag, F. and Koch-Nolte, F. (1998) "Endogenous relatives of ADP-ribosylating bacterial toxins in mice and men: potential regulators of immune cell functions", *J. Biol. Regul. Homeost. Agents* **12**, 53–62.

Kahl, S., Nissen, M., Girisch, R., Duffy, T., Leiter, E.H., Haag, F. and Koch-Nolte, F. (2000) "Metalloprotease-mediated shedding of enzymatically active mouse ecto-ADP-ribosyltransferase ART2.2 upon T cell activation", *J. Immunol.* **165**, 4463–4469.

Koch-Nolte, F. and Haag, F. (1997) "Mono(ADP-ribosyl)transferases and related enzymes in animal tissues. Emerging gene families", *Adv. Exp. Med. Biol.* **419**, 1–13.

Koch-Nolte, F., Duffy, T., Nissen, M., Kahl, S., Ablamunits, V., Leiter, E.H. and Haag, F. (1999) "A new monoclonal antibody detects a developmentally regulated mouse ecto ADP-ribosyltransferase on T cells: subset distribution, inbred strain variation, and modulation upon T cell activation", *J. Immunol.* **163**, 6014–6022.

Liu, Z.X., Azhipa, O., Okamoto, S., Govindarajan, S. and Dennert, G. (2001) "Extracellular nicotinamide adenine dinucleotide induces T cell apoptosis *in vivo* and *in vitro*", *J. Immunol.* **167**, 4942–4947.

Ludden, P.W. (1994) "Reversible ADP-ribosylation as a mechanism of enzyme regulation in procaryotes", *Mol. Cell. Biochem.* **138**, 123–129.

MacKenzie, A., Wilson, H.L., Kiss-Toth, E., Dower, S.K., North, R.A. and Surprenant, A. (2001) "Rapid secretion of interleukin-1beta by microvesicle shedding", *Immunity* **15**, 825–835.

Okamoto, S., Azhipa, O., Yu, Y., Russo, E. and Dennert, G. (1998) "Expression of ADP-ribosyltransferase on normal T lymphocytes and effects of nicotinamide adenine dinucleotide on their function", *J. Immunol.* **160**, 4190–4198.

Prochazka, M., Gaskins, H.R., Leiter, E.H., Koch-Nolte, F., Haag, F. and Thiele, H.G. (1991) "Chromosomal localization, DNA polymorphism, and expression of Rt-6, the mouse homologue of rat T-lymphocyte differentiation marker RT6", *Immunogen* **33**, 152–156.

Thiele, H.G., Haag, F. and Koch-Nolte, F. (1997) "Molecular cloning and characterization of the T-cell mono(ADP-ribosyl)transferase RT6. Relationships to other mADPRTs and possible functions", *Adv. Exp. Med. Biol.* **419**, 109–120.

van Parijs, L. and Abbas, A.K. (1998) "Homeostasis and self-tolerance in the immune system: turning lymphocytes off", *Science* **280**, 243–248.

Wang, J., Nemoto, E., Kots, A.Y., Kaslow, H.R. and Dennert, G. (1994) "Regulation of cytotoxic T cells by ecto-nicotinamide adenine dinucleotide (NAD) correlates with cell surface GPI-anchored/arginine ADP-ribosyltransferase", *J. Immunol.* **153**, 4048–4058.

Wang, J., Nemoto, E. and Dennert, G. (1996) "Regulation of CTL by ecto-nictinamide adenine dinucleotide (NAD) involves ADP-ribosylation of a p56lck-associated protein", *J. Immunol.* **156**, 2819–2827.

